# Optimizing automated characterization of liver fibrosis histological images by investigating color spaces at different resolutions

**DOI:** 10.1186/1742-4682-8-25

**Published:** 2011-07-14

**Authors:** Doaa Mahmoud-Ghoneim

**Affiliations:** 1 Physics Department, Faculty of Science, United Arab Emirates University, Al-Ain, UAE; 2 Biophysics Department, Faculty of Science, Cairo University, Giza, Egypt

## Abstract

Texture analysis (TA) of histological images has recently received attention as an automated method of characterizing liver fibrosis. The colored staining methods used to identify different tissue components reveal various patterns that contribute in different ways to the digital texture of the image. A histological digital image can be represented with various color spaces. The approximation processes of pixel values that are carried out while converting between different color spaces can affect image texture and subsequently could influence the performance of TA. Conventional TA is carried out on grey scale images, which are a luminance approximation to the original RGB (Red, Green, and Blue) space. Currently, grey scale is considered sufficient for characterization of fibrosis but this may not be the case for sophisticated assessment of fibrosis or when resolution conditions vary. This paper investigates the accuracy of TA results on three color spaces, conventional grey scale, RGB, and Hue-Saturation-Intensity (HSI), at different resolutions. The results demonstrate that RGB is the most accurate in texture classification of liver images, producing better results, most notably at low resolution. Furthermore, the green channel, which is dominated by collagen fiber deposition, appears to provide most of the features for characterizing fibrosis images. The HSI space demonstrated a high percentage error for the majority of texture methods at all resolutions, suggesting that this space is insufficient for fibrosis characterization. The grey scale space produced good results at high resolution; however, errors increased as resolution decreased.

## Background

Digital encoding of microscopic images has enhanced the value of histological analysis, allowing quantitative rather than only qualitative assessment, using image analysis and measurement methods [1,2]. Image analysis techniques can describe a histological section and assign digital patterns to one or more pre-defined categories, allowing histopathologists to refer to a consistent database of features collected from similar cases rather than relying on subjective human assessments of individual samples. However, the limitations of image analysis methods must be considered. In addition to the classical problem concerning artifacts in histological sections, difficulties related to image quality including noise, resolution, contrast and illumination should be controlled. The effect of these factors on digital histological images has not been fully investigated but there is growing interest in this area [3,4]. Automated approaches can be categorized as texture, object and structure -based analysis [2]. According to Kayser *et al*. [2], texture-based analysis is defined as grey value per pixel measure, and it is independent from any segmentation procedure. It results in recursive vectors derived from time series analysis and image features obtained by spatial dependent and independent transformations [2]. Object-based features are defined as grey value per biological object measured, and structure-based features rely on identifying structural patterns that characterize a structure.

This research concerns the elaboration of texture-based features from microscopic images using a method known in the literature as Texture Analysis (TA). TA is a digital image analysis method that was successfully applied to medical and histological images. TA contributes to tissue characterization by detecting pathological modifications and can be used to characterize the effect of a given treatment. For example, TA can be used for detecting the progression or regression of a disease [5], and for therapeutic follow-up of subjects that respond to treatment and those that do not [1]. Therefore, TA provides a wide range of pharmacological applications. Features extracted from clinical and experimental digital images are subjected to a classification process that orders input data into output classes. Usually, these classes are interpreted in terms of relevance to other histological or biochemical parameters. TA has a particular diagnostic importance when local heterogeneities are investigated or when the disease is subtle and hard to detect visually [6]. Owing to successful characterization of tissues at various levels of progression and protection [1,7], histopathologists became interested in utilizing TA in problematic diagnostic tasks, such as grade assessment (grading), which is usually limited by a large number of variables, sample size restrictions and sampling variability [8]. TA is a faster quantitative tool than conventional human-dependent methods that are time consuming and unlikely to be error-free [8]. The time factor is a crucial element in the choice of assessment method, particularly in clinical applications, where large numbers of patients are scheduled for routine scanning. Grading and other automated assessment tasks require the accuracy of TA to be improved to increase its diagnostic value.

Previous work by the author revealed that the microscopic section staining protocol can play a major role in TA of liver fibrosis, demonstrating that histological texture can differ according to the staining protocol used and due to chemical interactions between the dye and the cell/tissue components that cause staining to appear [7]. The staining protocol confers specific colors to different cell components; the colors vary in terms of intensity and saturation depending on the underlying chemical interactions. In conventional TA, the original multi-channel colored sections are transformed into the corresponding single channel of the grey scale [1]; therefore, the texture specific to a color channel is lost, and instead, the texture of the approximated single channel appears. However, the grey scale image has been considered sufficient for fibrosis characterization in previous studies [7], but for more sophisticated assessment tasks (such as grading), the approximation of colored images to the grey scale could result in the loss of valuable texture information embedded in the individual color channels. This information could be crucial for increasing the accuracy of this method.

Color is an intrinsic attribute that provides more visual information than the grey scale. There have been several attempts to incorporate color information into texture [9,10] but the choice of which color space is best for performing TA has received little attention [3]. Research concerning the human visual system suggested that the overall perception of color is formed through the interaction of a luminance component, a chrominance component and the achromatic pattern [11]. The luminance and chrominance components extract color information, while the achromatic pattern component concerns texture. There are two approaches concerned with incorporating color and texture: one considers that these are different characteristics and that each characteristic cues independently [9,12,13]; the other approach considers color and texture as a combined characteristic. These methods predominantly use the multi-channel versions of grey scale texture descriptors [9,11] and some studies have demonstrated that incorporating color into texture improves classification results [3,13,14]. RGB space (representing red, green and blue, respectively) is the most common format used for digital image display. Color texture features can be extracted from this space separately or from cross-correlation between two colors. It has been demonstrated that incorporating texture features from the RGB space could enhance the accuracy of classification [3,13]. HSI (representing hue, saturation and intensity, respectively), another color space, can be produced by applying special filters and can be inspiring for the human eye [3,14]. Attempts have been made to study image features of histological images in this space [3]. However, the effect of this space on TA of microscopic images of biological tissues remains unknown.

In this paper, the objective was to apply TA to histological images of normal and fibrotic liver from experimental animals and to investigate the effect of selecting the color space on the accuracy of texture classification when image resolution changed. The three color spaces used in this work were the grey scale, RGB and HSI.

## Methods

### Experimental procedures

The experimental procedures described herein were carried out during previous work published by our group [7]. In this experiment, 12 male Wister rats were randomly placed into two groups: Control (C, n = 5) and Fibrosis (F, n = 7). They were fed a standard pellet diet and tap water *ad libitum*, placed in polycarbonate cages with wood chip bedding under a 12 h light/dark cycle, and kept at a temperature of 22-24°C. The C group received an intra-gastric injection of corn oil (1 ml/kg) twice a week. Liver fibrosis was induced in the F group by intra-gastric injection of CCl_4 _(1 ml/kg body weight, 1:1 in corn oil). This treatment was carried out for eight weeks [7]. Immediately at the end of experiments, animals were sacrificed and the liver excised. Samples were collected, frozen in liquid nitrogen and stored at -80°C [7]. This experiment was conducted following the guidelines of the Animal Research Ethics Committee of United Arab Emirates University [7].

**Figure 1:**
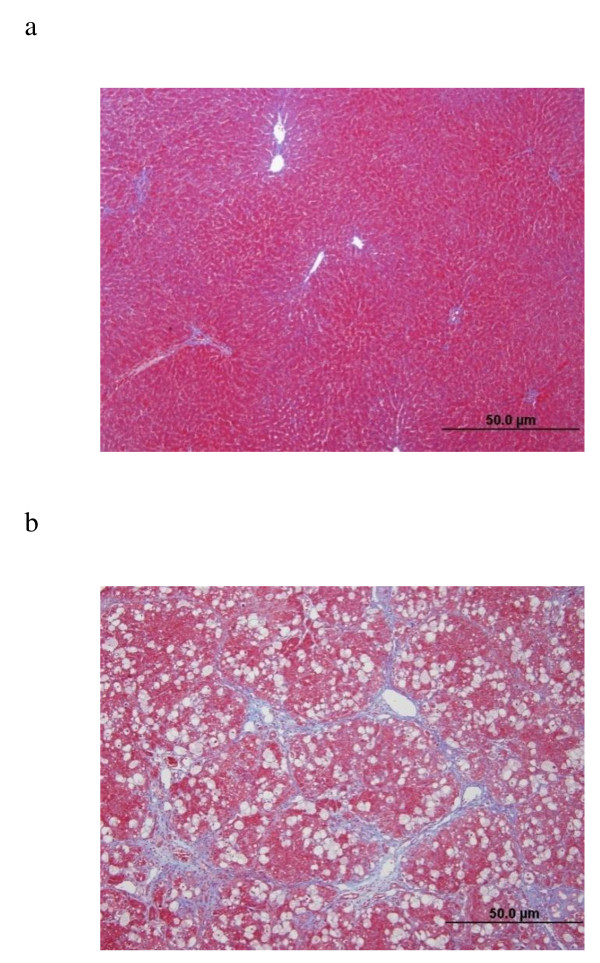
**Liver microscopic images**. Examples of liver microscopic images taken from (A) normal and (B) fibrotic tissues.

The presence of fibrosis was confirmed using histochemistry and histopathology [7]. Liver damage in the F group was assessed blindly on paraffin waxed sections stained for cellular and extracellular components using Masson's trichrome as described in Amin *et al*. [7]. In the current work, microscopic images of liver were taken and digitized using a Leica DMRB/E light microscope (Heerbrugg, Switzerland) and an Olympus camera, DP72. One microscopic image, clearly stained with no visible artifacts, was taken from each animal. Images from sections containing large blood vessels were avoided. Images were stored in Bitmap format (BMP) of 680 columns × 512 rows, 24 bit, true color and RGB pictures (Figure 1a, b). The liver sections of the C group had a normal histological appearance (Figure 1b). The fibrotic changes in sections from group F were visible by eye and were seen as strands of collagen deposition in the extracellular matrix (Figure 1b). More details concerning collagen quantification and other fibrosis related parameters for this experiment can be found in a previously published work [7].

Three categories of image resolution were studied for the C and F groups: (i) the images kept at original resolution indicated as "Full-resolution" images; (ii) resolution reduced to half of the original value so that the dimensions of the new image became 340 columns × 256 rows and indicated as "Half-resolution" images; (iii) resolution reduced to quarter of its original values so that the dimensions of the image became 170 columns × 128 rows and indicated as "Quarter-resolution" images. Each image was sub-divided into four equally sized non-overlapping regions of interest (ROIs), avoiding boundaries and small vessels, and outlining the hepatic structure with cells and the extracellular matrix. The total number of ROIs (sub-divisions) was 28 for the F group and 20 for the C group for each resolution category.

**Figure 2:**
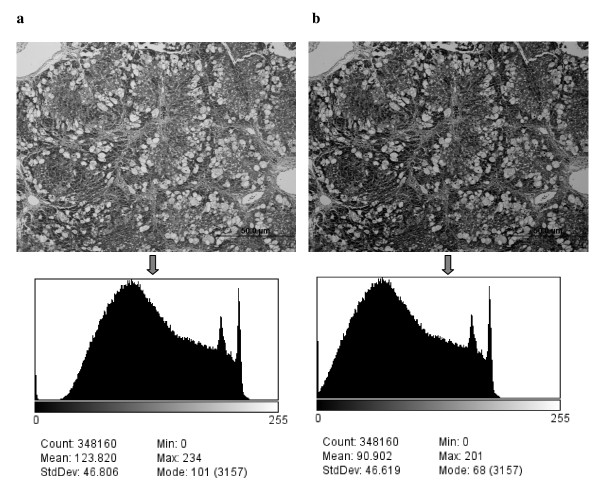
**Normalization example**. In image (a) the histogram occupies a certain range, giving a mean grey value of 123.8. The image (b) is the darker version of (a), giving a mean value of 90.9. The image (b) can be rescaled to (a) using the normalization process.

The illumination conditions or brightness settings under the light microscope can change from one slide to another for various reasons. This causes the grey scale histogram to shift to a different range; consequently, the comparison between textures from different images becomes inconsistent. In order to bring all images to the same range of grey scale a normalization (standardization) process should be carried out, with the aim of setting a standard mean value to all images and recalculating the grey scale in each image relative to this value; therefore, all textures become comparable. Accordingly, all ROIs were normalized to μ ± 3σ (where μ is the mean value and σ is the standard deviation of grey scale values in the image ROI) [4], the range obtained was then quantized to 7 bits (between grey values 1 and 128). An example is given in Figure 2, which presents two identical images of various brightness and the corresponding histograms. The histograms have similar profiles; however, the mean values are different as the histograms occupy different ranges. Normalization, as described above, solves this problem and removes dependency on pixel intensity mean value [4].

### Color spaces

As RGB images are composed of three channels (red, blue and green), each channel can be viewed individually as a grey scale layer with an intensity range between 0 and 255 in a standard 24 bit bitmap format (BMP). All RGB ROIs were converted into a single layer grey scale image by calculating the equivalent luminance value at each pixel using the formula:(1)

This projects the RGB space into grey scale, representing luminance only. The above technique is the most commonly used, but there are other techniques discussed in the literature [11].

Original RGB ROIs were converted to HSI space. The HSI space separates chromaticity and intensity information, thereby providing chromaticity measures independent of intensity [11]. This detaches the intensity component from the color information and reduces the effects of variable lighting. HSI space is closer to the human visual perception and understanding of color. H represents the visual spectrum of perceived colours, I represents the brightness of a colour and S refers to the amount of white light mixed with a hue. HSI can be represented by a cone shape, where H is located on the perimeter, S radiates from the centre outwards and I is located on the axis of the cone. For I and S, the minimum and maximum values are 0 and 1, respectively. Mathematical details concerning RGB to HSI conversion are detailed elsewhere [15].

### Texture analysis

Three TA methods were applied to ROIs for the three color spaces (grey scale, RGB, and HSI) and for the three resolution categories (Full-Resolution, Half-resolution, and Quarter -Resolution). These methods were co-occurrence matrix (COM), run-length matrix (RLM) and wavelet transform (WT).

### Co-occurrence Matrix

Co-occurrence matrix (COM) is the most widely used TA method in biomedical imaging [1,6]. It is a statistical method that depends on calculating the probability of finding a joint occurrence of a pixel of grey scale value *i *with another of value *j *within a predefined conditions of distance (*d*, *d *= 1, 2, 3, ...etc pixels) and orientation (*θ*, *θ *= 0°, 45°, 90°, 135°) [16]. Numerous parameters can be calculated from this matrix including angular second moment, contrast, correlation, entropy, sum of squares, inverse difference moment, sum average, sum variance, sum entropy, difference variance and difference entropy [16]. These quantitative descriptors are capable of elaborating texture characteristic features for a group of images and discriminating between two groups based on these features, directly or via mathematical recombination of features. Information concerning the performance and limitations of COM can be found in the literature [6,16]. In this work, the distance and direction were defined so that the position of *i *in an image matrix (Im) is Im(x, y) and that of *j *is Im(x, y+1) where x is the row value and y is the column value. These positions of *i *and *j *are known to produce COM within a distance *d *= 1 and angle *θ *= 0°.

### Run-length matrix

Run-length matrix (RLM) is a statistical TA method defined as the matrix *P_θ _(i, l) *which calculates the number of runs that exist in an image for a pixel of grey scale value *i *and length *l *in a direction *θ*. The angle *θ *can be 0° (horizontal), 90° (vertical), 45° or 135°. The statistical parameters derived from this matrix are short run emphasis, long run emphasis, run length non-uniformity, grey level non-uniformity and run fraction [6,16]. RLM provides information concerning the coarseness of a texture. If the image has predominantly long runs then the texture is coarse, while short runs indicate fine texture.

### Wavelet transform

Wavelet transform (WT) is a linear transformation that operates on a data vector whose length is an integer power of two, transforming it into a numerically different vector of the same length. WT is a tool that separates data into various frequency components using high-pass and low-pass filters, and then investigates each component with resolution matched to its scale. Therefore, a given function can be analyzed at various frequency levels [6]. In image analysis, the original image is sub-divided into smaller sub-images at different scales on which low and high pass filters are applied. The energy *E_n _*is a parameter calculated on the sub-images at scale *n*, and can be characteristic for a group of images. The main advantage of WT is the multiscale representation of the function.

### Feature selection using Fisher coefficient

Texture parameters calculated as described above from COM, RLM and WT on the grey scale ROIs were indicated by "greylevel"- scheme. Those which were calculated on the RGB space were called "RGB"-scheme. In the RGB-scheme, parameters calculated from one TA method, whether it was on the R, G or B channel, were pooled together as one set of texture descriptors. For example, all COM parameters that were calculated on R, G or B channels were re-grouped together as RGB-scheme on full-resolution images. Similarly, the "HSI"-scheme represents the pool that contains all the parameters that came from H, S, and I layers for each TA method at a given resolution.

Following texture parameters calculation, and prior to each classification test, the three most discriminating parameters (indicated as features) were selected using the Fisher (*F*) coefficient and used as a basis for subsequent class separation. A higher *F*-coefficient indicates that the classes are more likely to be separable using this parameter [17]. The aim of this step is to reduce the large number of calculated texture parameters to those which can be taken as features and expected to characterize the tissue in the classification process. As a general precaution, the number of parameters chosen for classification should not exceed the number of samples in each group to avoid over-performance of the classifier.

### Raw data classification

Classification was performed in a space composed of three coordinates where each axis corresponds to a feature. ROIs with similar texture features tend to cluster closer as a cloud of points within the same neighborhood. Classification using data as described above is an unsupervised approach, as each point clusters independently of the others and without pre-knowledge of the sample group or mathematical recombination. In this work, channel separation, texture analysis, feature selection, data classification and other image manipulation processes were performed using MaZda-B11 software (version 4.5, ^©^1999-2006) [16,17] and Matlab 7 (^© ^1984-2004, The MathWork, Inc.).

## Results and discussion

**Figure 3:**
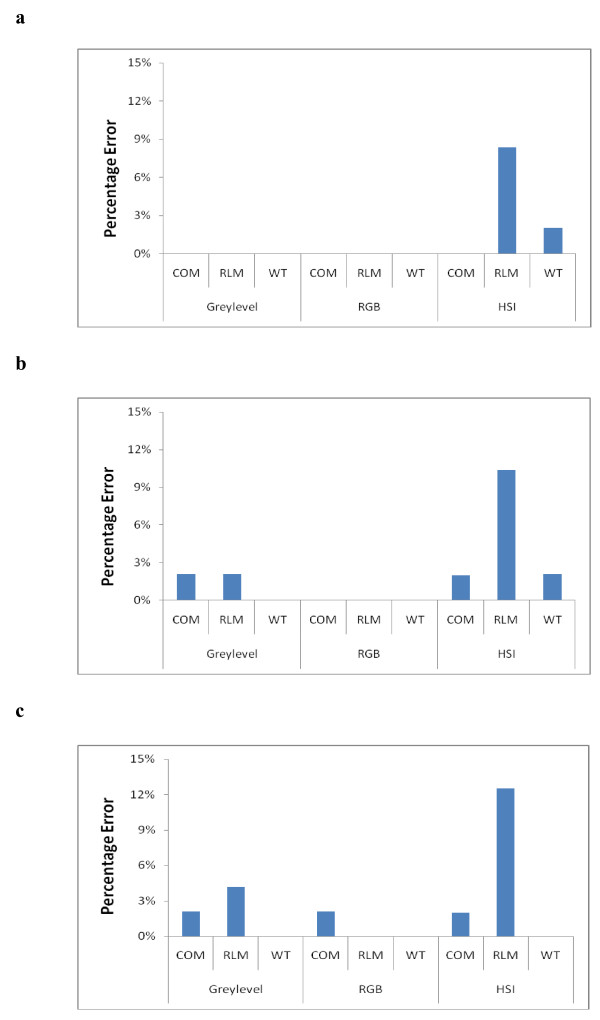
**Classification results**. Percentage error of texture classification in the C and F liver groups using the greylevel-, RGB- and HSI- schemes on: (a) full-resolution, (b) half resolution, and (c) quarter resolution images, using TA methods (COM, RLM, and WT).

The features selected by *F*-coefficient and used for classification are presented for the three resolution categories: Full-Resolution (Table 1), Half-Resolution (Table 2) and Quarter-resolution (Table 3). The classification results of C against F histological images based on texture features are presented as percentage error bars (histograms) for the resolution categories (Figure 3a, b, and 3c, respectively), and for each scheme using the three TA methods (COM, RLM and WT). The percentage error was calculated as the percentage ratio of misclassified samples to the total number of samples in one group.

**Table 1:** Texture features at full resolution

TA Method	Greylevel	RGB	HSI
COM	Sum of Squares	G_ Sum of Squares	H_ Sum Variance
	Sum Variance	R_ Sum of Squares	H_Correlation
	Sum Entropy	G_ Sum Variance	H_Inverse Difference Moment

RLM	Horizontal greylevel non-uniformity	G_ Horizontal greylevel non-uniformity	I_ Horizontal Run length non-uniformity
	Vertical greylevel non-uniformity	G_45° greylevel non-uniformity	I _Horizontal Fraction
	135°greylevel non-uniformity	G_135° greylevel non-uniformity	I _135° Run length non-uniformity

WT	*E_4_*	G_*E_4_*	I _*E_4_*
	*E_5_*	G_*E_5_*	S_*E_4_*
	*E_2_*	B_*E_4_*	I _*E_4_*

The texture features (parameters with the highest *F*-Coefficient) that discriminate between the C and F groups on greylevel-, RGB-, and HSI- schemes at full-resolution images, using TA methods:COM, RLM, and WT.

R_: Red, G_: Green, and B_: Blue channels. H_: Hue, S_: Saturation, and I _: Intensity. *E_s _*Energy calculated from the wavelets using various scales (*s*).

**Table 2:** Texture features at half resolution

TA Method	Greylevel	RGB	HSI
COM	Sum of Squares	G_ Sum of Squares	I _ Inverse Difference Moment
	Sum Entropy	R_ Sum of Squares	S_ Sum of Squares
	Sum Variance	G_ Sum Entropy	I _ Correlation

RLM	Vertical greylevel non-uniformity	G_45° greylevel non-uniformity	I _Vertical Long Run Emphasis
	Horizontal greylevel non-uniformity	G_ Horizontal greylevel non-uniformity	I _ Vertical Fraction
	45° greylevel non-uniformity	G_135°greylevel non-uniformity	I _ Vertical Run length non-uniformity

WT	*E_3_*	G_*E_3_*	I _*E_3_*
	*E_1_*	G_*E_3_*	I _*E_3_*
	*E_3_*	G_*E_4_*	I _*E_2_*

The texture features (parameters with the highest *F*-Coefficient) that discriminate between the C and F groups on greylevel-, RGB-, and HSI- schemes at half resolution images, using TA methods:COM, RLM, and WT.

R_: Red, G_: Green, and B_: Blue channels. H_: Hue, S_: Saturation, and I _: Intensity. *E_s _*Energy calculated from the wavelets using various scales (*s*).

**Table 3:** Texture features at quarter resolution

TA Method	Greylevel	RGB	HSI
COM	Sum Entropy	G-Sum Entropy	I _ Contrast
	Sum Variance	G-Sum of Squares	I _ Correlation
	Sum of Squares	G-Sum Variance	I _ Inverse Difference Moment

RLM	Horizontal greylevel non-uniformity	G_45° greylevel non-uniformity	I _ Inverse Difference Moment
	Vertical greylevel non-uniformity	G_ Horizontal greylevel non-uniformity	I _ Vertical Run length non-uniformity
	45° greylevel non-uniformity	G_ Vertical greylevel non-uniformity	I _ Vertical Long Run Emphasis

WT	*E_3_*	G_*E_2_*	I _*E_2_*
	*E_2_*	G_*E_1_*	S_*E_2_*
	*E_1_*	G_*E_3_*	I _*E_1_*

The texture features (parameters with the highest *F*-Coefficient) that discriminate between the C and F groups on greylevel-, RGB-, and HSI- schemes at quarter resolution images, using TA methods:COM, RLM, and WT.

R_: Red, G_: Green, and B_: Blue channels. H_: Hue, S_: Saturation, and I _: Intensity. *E_s _*Energy calculated from the wavelets using various scales (*s*).

In Figure 3a, which represents results for full-resolution images, no classification errors were evident using the greylevel- or RGB-schemes. However, the HSI-scheme had higher percentage errors with RLM and WT. The greylevel- and RGB-schemes were adequate to provide reliable texture features that maximized classification accuracy at this resolution. The remarkable increase in the percentage error for the RLM method with the HSI-scheme (8%) highlights the low performance of TA for this scheme and this method. At this resolution, the size of a pixel in the horizontal direction is approximately 0.255 μm of the actual histological sample size.

At half-resolution (Figure 3b), loss in classification accuracy was observed for greylevel- and HSI- schemes. The greylevel-scheme had a minor percentage error (approximately 2%) for COM and RLM. The HSI-scheme demonstrated a remarkable percentage error for RLM at this resolution (10%) but lower percentage errors for COM and WT. The RGB-scheme demonstrated zero percentage error for the three TA methods.

The quarter-resolution images (Figure 3c) represent higher percentage errors for the three schemes. The three schemes at this resolution had identical percentage errors for COM (2%). The greylevel- and HSI-schemes demonstrated a further increase in percentage error for RLM. However, the RGB-scheme had the lowest error among the schemes. The RGB-scheme demonstrated zero errors for RLM and WT. Comparing the three resolutions demonstrated that degradation of classification accuracy takes place as resolution decreases. Some color spaces were more susceptible to errors than others. The RGB-scheme was the most resistant to incidences of misclassification and produced more consistent results despite lowering resolution.

Obtaining acceptable results with RGB at low resolution refutes the idea that TA requires high resolution for good performance. The ability of achieving good classification results on low resolution images facilitates and reduces the time required for the process of TA, saves hardware space and therefore can be less expensive. In this respect, RGB space and the corresponding TA on the RGB-scheme provides the best accuracy-to-resolution compromise.

Although the texture parameters from the three RGB channels were pooled together, it was demonstrated that the majority of the discriminating parameters belong to the G (green) channel (Tables 1, 2, and 3). Discriminating parameters belonging to the R (red) or B (blue) channels rarely appeared as features (Table 1). This observation was consistent for the three TA methods and can be explained in terms of relevance to the staining protocol. The chemical interactions that occur between the staining substance and the cell or tissue component produce distinctively colored regions including pathologically dominant alterations (the extracellular collagen depositions in fibrosis). TA demonstrated an ability to characterize fibrosis on grey scale images and on specific color channels. The green channel was the most characteristic, revealing the majority of textural features (Tables 1, 2, and 3). Since this channel corresponds to the color of the extracellular collagen deposition, it can be concluded that collagen is the main characteristic for liver fibrosis that produces the most dominant texture, and this converges with histopathological findings in the literature [1]. It can be proposed that RBG space (and particularly the G channel) is more accurate than HSI results because the former is a true representation of light reflection from the tissue, while the latter is created by applying different filters, and yet it is a space approximated mathematically. Therefore, TA appears to be more efficient at characterizing pathological textural features from original spaces as demonstrated for RGB.

This research has demonstrated that the accuracy of TA results varies according to the color space used for the analysis and the resolution used. The RGB-scheme, corresponding to RGB space, produced better results than the greylevel- or HSI-schemes, particularly at low resolution. These findings are consistent with previous work concerning meningioma, where TA on RGB outperformed other color spaces owing to better discrimination on individual color channels [3]. Although the human eye can be more inspired by HSI space, this does not necessarily mean that this space would perform better for TA [3]. This study has demonstrated that HSI space was the poorest performer for TA. The superior results for the RGB space were predominantly because each color channel provided textural information that corresponded to a particular coloring effect of the staining dye specific for the most dominant pathological component. Therefore, RGB can characterize this component with higher accuracy within its color channel. When an RGB colored image is converted to grey scale by approximation methods, this results in individual channel information being undermined and errors occur. The results of this study emphasize two factors that should be considered when automated texture analysis and classification of liver microscopic images is targeted: firstly, texture and color are joint attributes and should be considered for classification in order to obtain increased accuracy of results, particularly when low resolution images are used; secondly, TA of individual color channels in an RGB space can highlight the pathological factor most useful for TA and therefore can be considered as important for further research concerning automated fibrosis assessment. TA is not the only automated method for pathology detection and characterization. Other methods, such as the theory of sampling [18] and newly developed tissue-based diagnosis methods [19], will increase the ability to obtain integrated information concerning biological tissues. Collectively, these automated methods can be used to produce a comprehensive base of knowledge for a disease, and this would facilitate the diagnosis at all stages of that disease.

## Conclusions

Color space affects the accuracy of classification of liver histological images at various levels of resolution. The grey scale is the conventionally used space for TA, but in this study RGB has demonstrated better results at low resolution, the ability to elaborate the pathological component most characteristic of fibrosis on histological images and emphasis on its corresponding channel. The results of this work could enhance the TA approach and highlights the factors that should be considered in future liver assessment challenging tasks using automated methods.

## Authors' contributions

DMG is the single author of this manuscript. The author carried out work procedures that included: acquiring data, applying methods, analyzing results, interpreting image analysis findings in relevance to biology, and writing the manuscript.

## Declaration of Competing interests

The author declares that they have no competing interests.
